# The diabetes distress experience from the perspective of adolescents and their parents

**DOI:** 10.3389/fcdhc.2026.1652578

**Published:** 2026-06-11

**Authors:** Raman Sangha, Natalie Grieve, Kyra Braaten, Simran Gill, Titilola Yakubu, Tom Warshawski, Tricia S. Tang, Trent Smith, Mary Jung, Elijah M. K. Haynes, Christine Voss

**Affiliations:** 1 Centre for Chronic Disease Prevention and Management, University of British Columbia, Kelowna, BC, Canada; 2 School of Health and Exercise Sciences, University of British Columbia, Kelowna, BC, Canada; 3 Faculty of Medicine, University of British Columbia, Vancouver, BC, Canada; 4 Interior Health, Kelowna, BC, Canada; 5 Interior Health, Kamloops, BC, Canada

**Keywords:** adolescent mental health, caregiver burden, emotional burden, psychosocial support, T1D management

## Abstract

**Objective:**

To explore sources of diabetes distress (DD) and resources for mental health support for adolescents with type 1 diabetes (T1D) and their parents/guardians.

**Methods:**

We recruited adolescents with T1D and parents of adolescents with T1D using an explanatory sequential mixed methods design. Participants completed measures of DD for adolescents (Problem Areas in Diabetes-Teen, PAID-T) or parents (P-PAID-T), followed by separate focus groups that elicited information on common concerns related to living with T1D, types of mental and emotional support accessed, and additional support sources desired. Data were analyzed using an interpretive description approach and collaborative analysis techniques.

**Results:**

Six adolescents and five parents participated. All adolescents reported high levels of DD (median (IQR) PAID-T score 71 (59-86), cutoff ≥ 44), while most parents reported low levels of DD (P-PAID-T score 46 (35-66), cutoff ≥ 54). Across all focus groups, five themes were identified: DD can cause burnout and indifference; T1D disrupts activities of daily living; More peer support is desired; Society is unaware of how T1D is managed; and Concern about future financial burden. The parent focus groups also identified: Parental concerns around transfer of management of responsibilities to their children.

**Conclusion:**

An improved understanding of the key sources of DD and how they compare between adolescents and parents will facilitate the design of more effective mental health support interventions for T1D.

## 1 Introduction

Type 1 diabetes (T1D) is a chronic autoimmune disease characterized by destruction of insulin-producing β cells in the pancreas and a consequent dysregulation of blood glucose levels (1). There is currently no cure for T1D. Effective management of this life-threatening condition requires exogenous insulin therapy, continuous blood glucose monitoring, carbohydrate counting and physical activity (2). T1D accounts for approximately 5% to 10% of all diabetes cases. Globally, 9.2 million people live with T1D, of which 1.85 million are < 20 years of age (3). In British Columbia (BC), Canada, there are about 2,200 children affected (4), with an estimated 220 new cases each year.

Adolescents with T1D commonly experience diabetes distress (DD), defined as the emotional burden attributable to the demands of disease management, as well as worries, fears, and concerns associated with living with this chronic condition (5). DD is associated with greater glycemic instability among adolescents with T1D (6). Prolonged elevated levels of distress can lead to increased average blood glucose levels and lower time in range (i.e., the proportion of time an individual’s glucose levels remain within the target range) (7). Moreover, adolescents with DD are also at increased risk of mood and anxiety disorders (8). In contrast, those with better physical and mental health report lower scores on DD screening questionnaires (9).

DD is common, with approximately one in three adolescents with diabetes reporting to experience significant DD (10). Yet, mental health resources for this population remain limited (5). Addressing DD could have a positive impact on the physical and mental health of adolescents with T1D, and this requires a comprehensive understanding of the contributing factors. Potential contributors include self-consciousness, self-stigma, daily management challenges, health care struggles, concerns about the future, and social isolation (11). These factors have been shown to negatively impact an individual’s self-management skills and social life, and thus intensify emotional distress (12).

In parallel, parents of adolescents with T1D can also experience DD as they assume additional management responsibilities, such as nighttime caregiving, which can have a considerable impact on caregivers' mood and health (13). Resources available to parents, which have typically been developed in urban rather than rural environments, are limited (14). Age at diagnosis, time since diagnosis, and the presence of comorbidities are all thought to impact the level of caregiver burden experienced by parents (14). Parental DD, particularly maternal DD, has been shown to significantly influence both depressive symptoms in parents and glycemic control in adolescents with T1D, highlighting the need to address DD in both groups (15, 16). Furthermore, discordance between parent- and child-reported distress impacts glycemic control and increases diabetes-specific family conflict (17).

Previous research has focused predominantly on addressing DD in adults with T1D (10, 18, 19) or in the parents of children with T1D (14, 20, 21). However, research and interventions focusing on DD in adolescents with T1D or in parents of adolescents with T1D are less common, despite adolescence being a time of increased vulnerability due to increasing independence and the looming transition from pediatric to adult services (22, 23). Existing research on DD in adolescents and parents of adolescents has largely concentrated on its prevalence, with some studies examining the effectiveness of digital interventions in mitigating symptoms (10, 11). However, there remains a lack of research exploring the factors that contribute to the exacerbation of DD symptoms in this population. This gap is particularly important given the complex and interdependent nature of parent-adolescent experiences, where distress may be shared, influenced by one another, or evolve within the dyadic relationship. Understanding this context is novel as it moves beyond individual perspectives, ultimately aiming to address the potential relational impact of DD and informing more tailored interventions. Additionally, many of these studies were conducted in urban areas and unable to account for the differences in health care access and resources for individuals with T1D in rural areas. Understanding the lived experiences of diabetes distress in rural contexts can also help to tailor interventions to meet unique needs, resources and constraints of rural living which may differ substantially compared to experiencing DD in urban contexts. Therefore, the aim of the present study was to explore the diabetes distress experiences of adolescents living with T1D, as well as those of parents of adolescents living with T1D, associated with managing this chronic condition in the context of the predominantly rural setting of the Interior of BC, Canada.

## 2 Methods

### 2.1 Study design and sample

**Figure 1:**
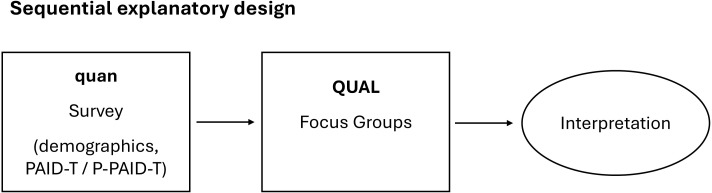
Sequential explanatory design.

This study had an explanatory sequential mixed methods design (**Figure 1**), whereby we first used a quantitative survey to gain insight into sample characteristics and DD prevalence, which was then followed by qualitative focus groups to explore in-depth the DD experiences of adolescents with T1D and parents of adolescents with T1D. Our approach was grounded in a pragmatic research paradigm, which focuses on selecting practical methods for data collection and analysis, with the ultimate goal of informing future interventions for adolescents living with T1D who are experiencing DD. Participants were recruited from two diabetes education centers (DEC) in the Interior region of BC, Canada, with one center being located in a mid-size city (population < 150,000) and the other being located in a small city (population < 10,000). Adolescents aged 13 to 19 years with T1D or the parents/guardians of adolescents with T1D were eligible for inclusion. The parents/guardians and adolescents did not need to be from the same family to participate in the study. It was estimated that approximately 60 adolescents would be eligible to participate across the two sites. Recruitment was led by clinical staff at each site whenever feasible in the context of busy clinical schedules and competing clinical priorities. Clinical staff provided a study flyer and instructed interested participants (adolescents with T1D and/or parents of adolescents with T1D) to reach out to study personnel directly. Study personnel then provided additional study information, answered any questions, and provided a link to complete electronic written informed consent and assent to participate in the study. Data collection took place between April 2022 and April 2023. First, participants completed an online survey (Qualtrics LLC, Provo, UT, USA) to collect information on sociodemographic characteristics, T1D-related health information, and level of DD. Then, online focus groups were conducted for adolescents and parents separately. Each focus group lasted approximately 60 minutes and was conducted with the same video conferencing platform (Zoom Communications, Inc., San Jose, CA, USA). Harmonized ethics approval was obtained from Interior Health Authority (2021-22-075-H) and the University of British Columbia (H21-02189; December 20, 2021).

### 2.2 Questionnaires

Adolescents were asked about their age, age at T1D diagnosis, gender, ethnicity, number of physician visits per year, insulin regimen, and use of technology for T1D management. Parents were asked similar questions about themselves and their adolescents with T1D, and were also asked to indicate their highest level of education, household income and whether they had extended health coverage. Diabetes Distress was assessed using the short versions of the Problem Areas in Diabetes-Teen Version (PAID-T) and Parents of Teens PAID-T (P-PAID-T) questionnaires (24). In this validated tool, participants rate their level of agreement on a 6-point Likert scale across 14 (PAID-T) or 15 items (P-PAID-T) in relation to the past month, with scores of ≥ 44 and ≥ 54 indicating high levels of DD on the PAID-T and P-PAID-T, respectively (24).

### 2.3 Focus groups

Focus group guides were developed to address our research objectives and were based on previous informal meetings with people with lived experience with T1D as well as clinical care providers for adolescents with T1D. Guiding and probing questions were defined to explore common and future concerns of living with T1D, experiences with DD, types of mental and emotional support accessed, and support they would like to be able to access (**Supplementary Material**). Overall, questions were exploratory in nature and designed to capture the diabetes distress experiences of adolescents and parents of adolescents living with T1D that are not adequately represented through conventional survey methods. Focus groups were held online (Zoom) separately for parents and adolescents. In total, one adolescent focus group and two parent focus groups were held to accommodate conflicting schedules. The same experienced qualitative researcher (TY) facilitated all focus groups. Focus groups were audio-recorded (Zoom) and transcribed using NVivo 12 (release 1.71; QSR International, Burlington, MA, USA), and identifying information was removed from transcripts before analysis.

### 2.4 Analysis

For questionnaire responses, descriptive statistics were calculated as frequencies for categorical data, and median (interquartile range, IQR) for continuous data, using R (version 4.4; R Foundation for Statistical Computing, Vienna, Austria). Interpretive description was chosen because it aligns with a pragmatic paradigm and supports the generation of clinically meaningful insights, identifying patterns across subjective experiences while accommodating individual variation, rather than aiming for data saturation (25). Focus group transcripts were analyzed thematically using NVivo 12 (QSR International). A purely inductive approach was used to generate interpretive descriptions of themes related to the experience of living with T1D (25). Coders employed a collaborative analysis technique: one researcher (RS) acted as the main coder and two others (NG and KB) independently provided critical and constructive feedback on their interpretation. Once all transcripts had been coded, overarching themes were inductively created by organizing similar codes together. Themes were then reviewed by all researchers and codes were defined.

### 2.5 Position statement

The focus groups were led by a researcher (TY) with experience in working with adolescents navigating the complexities of chronic illness management, but without first-hand knowledge of living with T1D. The interpretation of findings was led by an undergraduate psychology student (RS), who also did not have lived experience of T1D, but who was driven by a clinical interest in access to mental health supports for adolescents living with a chronic condition, particularly in rural settings.

## 3 Results

### 3.1 Sample characteristics

Six adolescents (4 girls, 2 boys; median age 17 years, IQR 17–19 years) enrolled in the study (approx. 10% recruitment rate). All six adolescents participated in the same single focus group. Surveys were available for only five adolescents as one participant was non-responsive to repeated requests to complete the survey, both prior to and following the focus group. Six parents/guardians enrolled in the study (6 women; median 51 years, IQR: 48–54 years), all of whom identified as mothers of adolescents with T1D. All six parents completed the survey, but only five participated in the focus groups due to scheduling conflicts. The mother who did not participate in the focus group did not experience DD and did not meaningfully differ in terms of sample characteristics from focus group participants. Two separate parent focus groups were hosted to accommodate different schedules. Detailed sample characteristics of focus group participants in terms of participant demographic information and adolescent T1D-specific information are provided in **Table 1**. Based on the (P-)PAID-T scores, all adolescents experienced significant DD, while just one parent experienced DD.

**Table 1:** Sample characteristics of focus group participants.

	Adolescents (*n* = 6)	Parents/guardians (*n* = 5)
Participant Age (years)	17 (17–18)	48 (47–53)
Participant Gender, *n* (%)		
Girl/Woman	4 (67%)	5 (100%)
Boy/Man	2 (33%)	0 (0%)
Non-binary	0 (0%)	0 (0%)
Participant Race/Ethnicity, *n* (%)*^†^		
White	4 (67%)	5 (100%)
Chinese	1 (17%)	0 (0%)
Métis	0 (0%)	1 (20%)
Missing	1 (17%)	0 (0%)
Adolescent Age at T1D Diagnosis (years)*	10 (4–15)	11 (7–14)
Adolescent T1D Management, *n* (%)*		
Insulin therapy		
Pump	4 (67%)	4 (80%)
Multiple daily injections	1 (17%)	1 (20%)
Missing	1 (17%)	0 (0%)
Glucose Monitoring		
Continuous Glucose Monitor	5 (83%)	4 (80%)
Glucometer	0 (0%)	1 (20%)
Missing	1 (17%)	0 (0%)
Primary Diabetes Clinic Setting, *n* (%)		
Mid-size City (population < 150,000)	4 (67%)	3 (60%)
Small-size City (population < 10,000)	2 (33%)	2 (40%)
Physician Visits (per year)		
1	1 (17%)	0 (0%)
1–2	0 (0%)	2 (40%)
3–4	3 (50%)	3 (60%)
> 5	1 (17%)	0 (0%)
Missing	1 (17%)	0 (0%)
Participant PAID-T or P-PAID-T score*	71 (59–86)	46 (35–66)
Participant Diabetes Distress, *n* (%)*		
Yes	5 (83%)	1 (20%)
No	0 (0%)	4 (80%)
Missing	1 (17%)	0 (0%)
Highest level of education, *n* (%)		
Some college or certification	–	2 (40%)
Graduate or Professional Degree	–	3 (60%)
Total household income, *n* (%)		
C$70,000 to C$99,999	–	1 (20%)
> C$100,000	–	4 (80%)
Extended health coverage, *n* (%)		
Yes	–	4 (80%)
No	–	1 (20%)

Data are median (interquartile range) or n (%).

*Data available for n=5 adolescents due to non-completion of the survey by one adolescent focus group participant.

^†^ might exceed 100% as participants could select multiple options.

PAID-T, Problem Areas in Diabetes-Teen Version; P-PAID-T, Parents of Adolescents with Diabetes PAID-T; T1D, type 1 diabetes.

### 3.2 Focus groups

**Figure 2:**
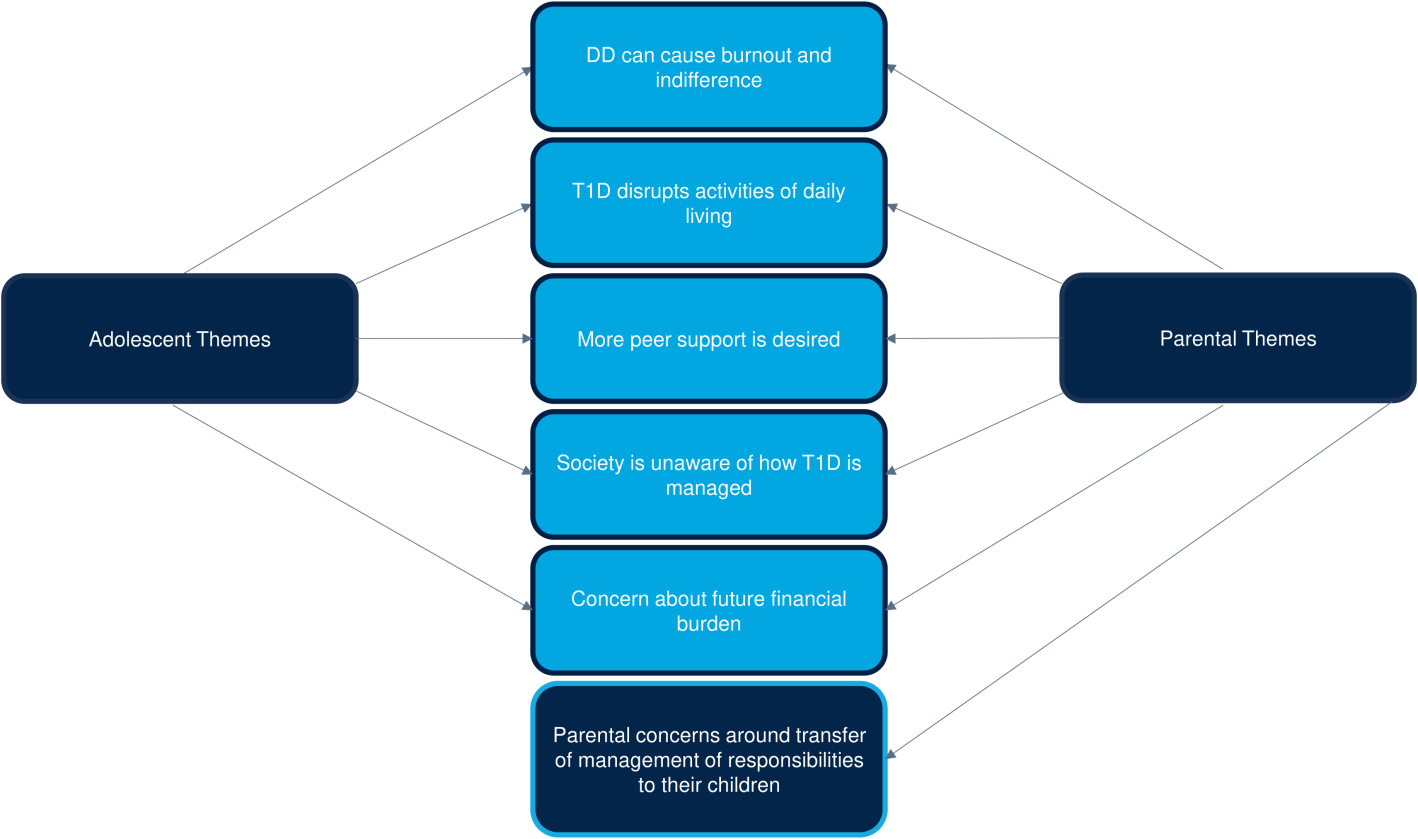
Themes related to diabetes distress in adolescents with type 1 diabetes and their parents/guardians.

Three focus groups were held in total. All adolescents (n=6) participated in the same single focus group, whereas two separate parent focus groups were hosted (n=2 and n=3) to accommodate conflicting schedules. Both adolescent and parent groups shared similar concerns about the experiences of adolescents living with T1D. The adolescent focus group identified five themes: DD can cause burnout and indifference; T1D disrupts activities of daily living; More peer support is desired; Society is unaware of how T1D is managed; and Concern about future financial burden. The same five themes were also identified in the parent focus group, along with an additional theme: Parental concerns around transfer of management of responsibilities to their children. A visual overview of themes, highlighting the overlap across adolescent and parent samples, is provided in **Figure 2**. **Table 2** provides supporting adolescent and parent quotes for each theme.

**Table 2:** Sample quotes representing each focus group theme.

Adolescents	Parents
DD can cause burnout and indifference	
*“I cannot just relax and enjoy doing things. It’s always an extra thing that I’m constantly worrying about, which just elevates my already very high levels of stress in my normal life … sometimes I just get so angry that I have to deal with this like I just get mad at the universe, and … the injustice, pisses me off so much, especially when my technology doesn’t work.” (Adolescent 4)*	*“Just like literally physically tired … the tank is empty and no more gas left. There are Saturdays where I just check out all day … it’s an exhausting feat, both mentally and physically. If you’re not thinking about it, you’re not sleeping over it because you’re getting up, and our son is…16 still—hypoglycemic unaware when he’s sleeping. He doesn’t rouse from lows, so we go up and sugar him up.” (Parent 4)*
T1D disrupts activities of daily living	
*“I feel like I’m constantly expected to be more prepared than everybody else … I want to stay alive, I got to be on it all the time, 24/7.” (Adolescent 4)*	*“I constantly worry about it. Yeah, [his] numbers are high all the time and I worry about his extremities, I worry about his kidneys. I worry about … just not going to into adulthood [and] self-managing appropriately. So, I very much worry about what that’s going to mean for him down the road.” (Parent 1)*
More peer support is desired	
*“I would say I’d definitely agree with like having in-person support even having like having that peer support of people who are type 1 is so important like this is one thing like getting to sit down and talk to everyone who is type 1.” (Adolescent 6)*	*“Maybe it would be nice once for all to have other parents to talk to you. However, not the parents of children who do their diabetes perfectly. More like the ones whose kids are imperfect … whose got the higher A1cs.” (Parent 3)*
Society is unaware of how T1D is managed	
*“I had a brief job with Canadian Pacific Railway and I had to talk to the superintendent of the entire region where I was working to get an exception for this … After he told me it was okay, they suddenly they stopped me one day while I was training, and they took me off the job, and they said, ‘Look, Transport Canada has said that you cannot have this. So, if you want to keep working, you need to just not have this at all … it’s a distraction thing … I know there’s the devices like the flash glucose monitoring one … you could just have that little receiver … That would be fine, that’s a medical device. This is not a medical device.’” (Adolescent 2)*	*“However, it is disappointing how little people are aware of what actually, what these individuals go through, and then also that they can make it easier, they can make it hard and making it hard just sucks for everybody.” (Parent 4)*
Concern about future financial burden	
*“The insurance thing freaks me out a bit, too, because in my dream life, I really wanted to be self-employed or employed by—I want to be an author. So that’s kind of a project-by-project basis thing, and that just means living is going to be more expensive if I can’t find another job that has good insurance or be successful enough by age 25 to be supporting myself on that, or like trying to have some other job on the side like I don’t know.” (Adolescent 4)*	*“She shares her concerns about, how am I going to afford this when I’m older? She’s concerned, you know, when she is no longer on our medical. What if she doesn’t have a job that has medical. It’s not cheap. I mean 3 months, for the Dexcom is like $1,200. Throw in the Omnipod and it’s a lot of money. So, she’s worried about that. At 14 she’s worried about how she’s going to pay for this disease when she’s 19.” (Parent 3)*
Parental concerns around transfer of management of responsibilities to their children	
*N/A*	*“In addition, when I say that it’s just me and my husband, he doesn’t let anybody else administer insulin. So, we’ve been very, very bound by his diabetes and his additional needs over the years.” (Parent 1)*

#### 3.2.1 DD can cause burnout and indifference

Some adolescents expressed strong negative emotions, such as anger and distress, in relation to their T1D experience (**Table 2**). They reported feeling burned out due to the prolonged excessive stress associated with managing their T1D. Having to repeatedly explain their diagnosis to others contributed to these feelings of burnout. Most adolescents reported that the combination of distress, anger, and burnout negatively impacted their overall mental health, resulting in a struggle to be optimistic about their future.

“That was kind of a moment where I realized that I’m literally … like, it’s living with a serial killer for a roommate.” (Adolescent 1).

The emotional impact of living with T1D was not monolithic. When asked whether they had issues with peers or had future concerns due to their diagnosis, one adolescent expressed indifference toward their circumstances.

“I mean, most of my peers are people I’ve been at school with since I got diagnosed, so it’s not a big difference.” (Adolescent 5).

“Did anyone treat you differently, because you have to, you know, explain to everyone? Were you treated differently?” (Interviewer).

“I didn’t see it really; I didn’t get anything like that toward me.” (Adolescent 5).

In the parent focus group, parents initially discussed their perception of the emotional impact of T1D on their children, and how their children coped with their T1D-related struggles. While adolescents often described feelings of anger and distress surrounding their diagnosis, parents’ emotional responses were more characterized by anxiety and fear. Parents also expressed strong negative emotions regarding their child’s struggles and potential future challenges related to T1D. They described feeling burnt out due to the immense amounts of time and emotional energy spent to support management of their child’s condition (**Table 2**). Parents also noted that continuous rumination of these thoughts further exacerbated their worries and had a negative impact on their mental health. It is noteworthy, however, that parents also voiced more optimism for their child’s future due to advances in technology related to T1D care, which may better aid in their child’s management of T1D.

“I still feel very optimistic that—with the improvements in technology, the access to some amazing health care professionals—that our kids are gonna live long and relatively healthy lives. So far, we’re doing well … I have to admit, I am cautiously optimistic that we’re not going to be faced with any really awful things going forward.” (Parent 2).

#### 3.2.2 T1D disrupts activities of daily living

Adolescents discussed their daily struggles with managing their T1D and how their T1D care interferes with many of their day-to-day activities, such as participating in sports and other social activities. Adolescents stated that the unpredictability of T1D requires them to be prepared for emergent challenges that create social and travel difficulties.

“It makes sports harder. It makes exercise harder. It makes hanging out with friends harder because literally, I’m bringing my scale with me to weigh chips like a loser at the party.” (Adolescent 4).

Furthermore, some adolescents reported that their overall quality of life has been negatively affected due to the unpredictability and complications that may arise with T1D (**Table 2**). Other adolescents, particularly those who were diagnosed at a younger age, indicated that diabetes has not greatly disrupted their ability to live a “normal” life.

Parents also discussed the struggles associated with T1D care due to the unpredictability and constant preparation required. They worried about their child’s T1D management during life transitions, such as moving for university, consuming alcohol, and driving.

“I’m worried about the whole eating thing when she goes on campus like how [is] she going to do that? Walk around with a scale? She’s not going to do that. She’s going to wing it. She’s gonna guess with everything and I am worried.” (Parent 5).

Parents also expressed concern about present and future complications or conditions that could negatively impact their child’s T1D management (**Table 2**). This was a major concern for one parent of a child with autism—a condition that impacts this particular child’s ability to manage their T1D independently. As this child is heavily reliant on their parents for T1D care, their attendance at school is minimal, making it challenging to follow a daily routine. More broadly, parents tended to emphasize worries about how such challenges interrupt daily activities and long-term functioning, whereas children often described these disruptions in terms of immediate frustration rather than concern about their broader impact.

#### 3.2.3 More peer support is desired

Adolescents discussed the support they received from peers, family, and mental health professionals. Although most stated that they felt adequately supported by family, some adolescents remarked that their parents can actually be unhelpful (e.g., miscarried helping). Nonetheless, adolescents stated that they acknowledged the positive intention behind the support from their parents and did not resent them for it. With regard to their peers, they stated that others without T1D do not understand the scope of their chronic condition and reported feelings of isolation from those who cannot relate to the experience of living with T1D. Many adolescents stated that they would like to have more access to peer support groups, either in-person or virtually, to connect with other adolescents with T1D and share their struggles and experiences (**Table 2**).

“But there’s something different about being in-person and being able to look at someone and be right there and talk to them. So, just having like that opportunity to meet with other Type 1’s in your area and talk to them and really connect about that, because it’s not easy to do.” (Adolescent 6).

Parents made similar statements about feeling isolated from parents of children without T1D. They expressed a desire to be part of a group, either in-person or virtually, with other parents that understand the parental struggles associated with T1D. Specifically, many parents stated that there is a lack of community for parents, especially for parents of children whose T1D is not “perfectly” controlled (**Table 2**). To facilitate community, parents expressed a preference for group-based support over individual support for themselves. With regard to social support and mental health support for adolescents, parents stated that they ensure their child is aware of the professional and familial support available to them, although they did not always recognize that some of their own efforts to provide support could be perceived by adolescents as unhelpful (e.g., miscarried helping). Parents also discussed the resources available to their adolescents that aid in the management of T1D. Resources ranged from the availability of technology used in managing T1D to access to health care professionals and other social groups for questions regarding T1D. Some adolescents stated that they received professional help from a licensed mental health practitioner. The reasons for doing so were not always related explicitly to diabetes although topics related to the emotional aspect of diabetes were discussed. With regard to support from other practitioners involved in their diabetes care, adolescents stated that they would like to receive more emotional support surrounding their struggles with management.

“I just wish doctors were less condescending. Like, my diabetes team is great, but my doctor sometimes is really frustrating because it’s like, I am doing my best right now, and (it) is not helpful for my mental health if you are only pointing out when I do something wrong, or when I don’t know something and never tell me what I’m doing great!” (Adolescent 4).

Most parents also stated that their child has received mental health support from a licensed practitioner for reasons unrelated to diabetes. One parent mentioned that their child works with a psychologist experienced in diabetic care that has extensive knowledge of what a T1D diagnosis entails. Some parents shared their experiences accessing mental health support but described feeling inadequately supported due to a lack of understanding about the emotional impact of chronic diseases. Parents also reported mixed preferences for in-person versus virtual mental health support.

#### 3.2.4 Society is unaware of how T1D is managed

Many adolescents reported that T1D draws unwanted attention due to a lack of knowledge and negative perceptions of others surrounding T1D care. Adolescents also acknowledged their own previous lack of knowledge before their diagnosis. Many adolescents described the inequities they faced when managing their diabetes, including not being allowed to have their pump during exams or on the job at work (**Table 2**). Adolescents stated that many people confuse T1D with T2D, resulting in unwanted comments about their weight or eating habits.

“I feel the eyes, because obviously people are going to stare like it’s something they’ve never seen before. Just getting the eyes and the questions, and like the ‘Oh, but you’re not fat.’ That’s the biggest one I get, and it’s the worst.” (Adolescent 6).

Parents also discussed the inequities their children face in the classroom due to others being unfamiliar with what T1D care entails. Many parents echoed similar statements made by adolescents regarding the negative perceptions others have about a T1D diagnosis (**Table 2**). To combat stigma, one parent explained that they put in extra effort to raise awareness and educate others about T1D and the care it requires. They found that by doing this, they receive fewer questions and negative perceptions about their child’s T1D.

#### 3.2.5 Concern about future financial burden

All adolescents were concerned about their ability to afford the expenses related to their T1D when they will no longer be covered by their parents’ extended insurance. Adolescents were aware of the costs associated with their T1D care, and many were thinking ahead to ensure that they will be able to cover these costs in the future. For example, one adolescent shared how they might not pursue a career they are passionate about because of the lack of financial stability needed for someone living with T1D (**Table 2**). Another adolescent was concerned about finding a job that will allow them to manage their T1D care financially.

“My biggest worry right now is finding a job, because I’m in school and trying to plan my future to make sure that, when I graduate, I have to be in the right field to have … to get a job, to get insurance right away, or else I can’t live in my luxury of having an Omnipod and a Dexcom.” (Adolescent 6).

Parents acknowledged the future affordability concerns expressed by their adolescent children (**Table 2**). However, when asked about general issues of concern related to T1D, parents themselves did not express concerns about their child being able to afford T1D care in the future.

#### 3.2.6 Parental concerns around transfer of management of responsibilities to their children

Parents were ambivalent toward giving their child more independence managing their T1D as they enter adulthood. Many parents shared that they know their child is capable of managing their condition themselves, but struggle with letting go of that responsibility completely.

“My husband is very consumed by it. I’m trying to find the sweet spot about giving [my son] some, you know, independence. However, then, at the same time, we’re still texting him like, ‘Did you bolus? Are you going to bolus? How much are you going to bolus? Just when are you going to bolus?’” (Parent 4).

The parent caring for a child with autism identified an additional concern—the prolonged support their child could need due to the complexities associated with this co-occurring condition. This parent stated that their child is dependent on them for administering insulin, as their child is not comfortable using an insulin pump or a continuous glucose monitor. Therefore, granting more independence is a major challenge in this case (**Table 2**).

## 4 Discussion

This study utilized online surveys and focus groups to better understand sources of DD in adolescents and parents living in rural areas of BC, Canada. Our findings suggest that both adolescents and parents experience distress due to the emotional toll of a T1D diagnosis, disruptions to their daily lives, lack of support from others (peers, teachers, health care professionals), negative perceptions of T1D, and limited knowledge about T1D. These findings extend the literature by highlighting the interconnected nature of DD experiences, suggesting that DD is not solely an individual phenomenon and is shaped by dyadic and rural context. It advances the field beyond predominantly individual approaches and provides insight to inform interventions that could target both parents and adolescents in rural areas with limited resources and unique needs. In previous studies, young adults with T1D reported similar sources of distress, leading to feelings of stigmatization, which thus discouraged them from seeking help (11). Studies have found high levels of caregiver burden among parents of children with T1D due to the additional physical and emotional support required (14). Adolescents also shared significant concerns about future costs of managing their T1D, which were likely influenced by anticipated life transitions, such as starting university or embarking on future career paths. Given the ever-changing nature of coverage for essential T1D medications and devices, it is understandable that adolescents on the cusp of adulthood would be concerned about their future financial status. It is noteworthy that PAID-T does not directly query adolescents about present or future financial concerns, which are generally entwined with educational and career pursuits.

Interestingly, adolescent distress over future T1D-related costs was not at the forefront of their parents’ worries. Instead, parents expressed concern about transferring responsibilities related to T1D care onto their child as they enter adulthood. While it is the adolescent who has T1D, it is clear that the condition affects loved ones in both similar and different ways.

Both adolescents and parents discussed the strong negative emotions felt due to present and future T1D struggles. The distress conveyed by adolescents aligned with the higher PAID-T scores observed in the adolescent sample as well as with previous findings (5, 10). In contrast to other studies (26), however, the P-PAID-T score achieved the threshold for DD in only one parent. This difference may have been attributable to the optimism of parents about future advances in technology that will improve diabetes management for their children.

In addition, parents who administer insulin were reported previously to exhibit higher levels of caregiver burden (14). This might explain the lower P-PAID-T scores among parents, as most adolescents in our sample used insulin pumps. Many adolescents also stated that diabetes majorly affects their daily lives. However, adolescents diagnosed at a younger age did not echo this statement. Although our qualitative findings revealed associations between duration of diabetes and distress scores in teens, previous studies indicated no such relations (7, 27). Therefore, as stated in the focus groups, adolescents who have had diabetes for longer may feel better adjusted to their diabetes care. However, although it may not markedly impinge on their activities of daily living, T1D can still impact their mental health indirectly.

Stigma surrounding a diabetes diagnosis impacted the willingness of parents and adolescents to reach out for peer support. Many adolescents stated that they felt stigmatized due to others mistaking T1D for T2D, resulting in negative comments about weight or eating habits and implying that they are at fault for having T1D. This may induce feelings of shame and embarrassment, impacting adolescents’ self-management of care, and lead to avoidance of social activities and high levels of emotional distress (11, 12). Furthermore, adolescents stigmatized due to a T1D diagnosis have been reported to show a lower quality of life and higher DD scores (12). Previous research indicated that adolescents might hesitate to seek emotional support from others without T1D to avoid stigma that would negatively impact their mental and physical health (14). Most parents remarked that there is a lack of community for parents with T1D children, especially for those whose T1D management is suboptimal. Indeed, feeling judged might cause parents to withdraw from groups when their child is struggling with T1D management (**Table 2**), especially in rural settings where fewer peers are likely to share the experience. Therefore, virtual peer support interventions are being explored, and preliminary findings suggest that these interventions are both feasible and acceptable to parents of children with T1D (28).

Most adolescents in this sample had received professional mental health support, although this support did not center on issues related to diabetes. Despite receiving such support, all adolescents in our sample exhibited high levels of DD, suggesting that professional mental health support is not sufficient to alleviate DD, possibly because these practitioners lacked understanding of—or experience with—adolescents with T1D. The adolescents expressed frustrations with other health care professionals (i.e., primary care providers) involved in their care due to a lack of empathy when deviations from optimal management occurred. Other studies have suggested that health care professionals tend to disregard emotional struggles with T1D in teens and young adults (11). In addition, they might not be equipped to address the emotional aspects of diabetes due to time constraints, limited resources, and lack of experience in screening and addressing DD (29). The focus on physical outcomes over psychosocial outcomes in the health care system could contribute to patients feeling frustrated and unsupported in their diabetes care (29). Future implications may include incorporating empathy training to help manage diabetes-related distress, which could in turn support more favorable outcomes and improve long-term T1D management. Parents also stated that they felt inadequately supported when accessing mental health support due to a lack of knowledge among health care providers about the struggles associated with having a child with T1D. Instead, both adolescents and parents expressed a desire for more peer support from other adolescents with T1D or other parents with T1D, respectively, who can understand and relate to struggles associated with managing this chronic condition. Therefore, peer rather than professional support may be more important to adolescents and parents to facilitate feelings of belonging missing from interactions with mental health professionals or other professionals involved in their care. This study revealed interest among adolescents with T1D and their parents in both virtual and in-person forms of peer support. These findings may inform the interventions aimed at alleviating DD among adolescents and parents of adolescents with T1D in rural communities, particularly through virtual modalities that facilitate peer connections based on lived experience.

This study had several limitations that need to be considered when interpreting findings. First, the sample size for both adolescent and parent groups was relatively small. We experienced recruitment challenges, as clinical staff in busy diabetes clinics had limited capacity to recruit participants. Recruitment may also have been hindered by the research focus on diabetes distress, as stigma, discomfort discussing distressing experiences, and concerns about disclosure are well-documented barriers to adolescent engagement in mental health research (30). Our relatively low recruitment rate of approximately 10% could suggest potential self-selection bias, with study participants more willing or able to engage in discussions about DD. Regardless, smaller focus group sizes, such as ours, are advantageous for adolescent samples in the context of sensitive topics to ensure that every voice can be heard (31). However, it is possible that the focus group format could have impacted the experiences adolescents were willing to share in a group setting, as adolescents have been shown to feel more comfortable sharing emotional experiences with DD individually with a research assistant through semi-structured interviews (32). We did not have access to clinical data regarding T1D-related hospital admissions or historic glycemic control patterns, which could have provided additional context for the interpretation of our participants’ experiences. While our study was not designed to generate representative or generalizable findings, the small, relatively homogenous sample, which comprised exclusively of White and fairly affluent women in the parent sample, may have limited the breadth of experiences and perspectives captured and may limit the transferability of findings to more diverse populations.

## 5 Conclusions

These findings emphasize the need for interventions to improve the psychological well-being of adolescents with T1D during their pivotal developmental years, especially in rural areas where there are likely to be fewer resources and a lack of available support. Interventions should also consider the needs of their parents, including parental DD, to enhance their ability to support their child’s well-being. Recommended strategies include expanding access to group-based T1D support and increasing knowledge among rural health care professionals of the psychosocial challenges faced by adolescents with T1D. 

## Data Availability

The datasets presented in this article are not readily available because of ethical restrictions. Requests to access the datasets should be directed to CV, christine.voss@ubc.ca.

## References

[1] Pugliese A . Pathogenesis of type 1 diabetes. In: Bonora E DeFronzo RA , editors.Diabetes epidemiology, genetics, pathogenesis, diagnosis, prevention, and treatment. Springer International Publishing, Cham (2018). p. 141–79.

[2] Halperin IJ Wicklow B Amed S Chambers A Courage C Cummings E . Glycemic management across the lifespan for people with type 1 diabetes: A clinical practice guideline. Can J Diabetes. (2025) 49:5–18. doi: 10.1016/j.jcjd.2025.01.001 40155190

[3] Ogle GD Wang F Haynes A Gregory GA King TW Deng K . Global type 1 diabetes prevalence, incidence, and mortality estimates 2025: Results from the International Diabetes Federation atlas, 11th edition, and the T1D index version 3.0. Diabetes Res Clin Pract. (2025) 225:112277. doi: 10.1016/j.diabres.2025.112277 40412624

[4] Fox DA Islam N Sutherland J Reimer K Amed S . Type 1 diabetes incidence and prevalence trends in a cohort of Canadian children and youth. Pediatr Diabetes. (2018) 19:501–5. doi: 10.1111/pedi.12566 28857360

[5] Morrissey EC Casey B Dinneen SF Lowry M Byrne M . Diabetes distress in adolescents and young adults living with type 1 diabetes. Can J Diabetes. (2020) 44:537–40. doi: 10.1016/j.jcjd.2020.03.001 32507646

[6] Albright D Wardell J Harrison A Mizokami-Stout K Hirschfeld E Garrity A . Screening for diabetes distress and depression in routine clinical care for youth with type 1 diabetes. J Pediatr Psychol. (2024) 49:356–64. doi: 10.1093/jpepsy/jsae016 38647266 PMC11098041

[7] Inverso H LeStourgeon LM Parmar A Bhangui I Hughes B Straton E . Demographic and glycemic factors linked with diabetes distress in teens with type 1 diabetes. J Pediatr Psychol. (2022) 47:1081–9. doi: 10.1093/jpepsy/jsac049 35656859 PMC9801711

[8] Younes ZMH Abuali AM Farooqi MH Hassoun AAK . Prevalence of diabetes distress and depression and their association with glycemic control in adolescents with type 1 diabetes in Dubai, United Arab Emirates. Pediatr Diabetes. (2021) 22:683–91. doi: 10.1111/pedi.13204 33745208

[9] Stahl-Pehe A Glaubitz L Bächle C Lange K Castillo K Tönnies T . Diabetes distress in young adults with early-onset type 1 diabetes and its prospective relationship with HbA1c and health status. Diabetes Med. (2019) 36:836–46. doi: 10.1111/dme.13931 30761589

[10] Hagger V Hendrieckx C Sturt J Skinner TC Speight J . Diabetes distress among adolescents with type 1 diabetes: A systematic review. Curr Diabetes Rep. (2016) 16:9. doi: 10.1007/s11892-015-0694-2 26748793

[11] Balfe M Doyle F Smith D Sreenan S Brugha R Hevey D . What's distressing about having type 1 diabetes? A qualitative study of young adults' perspectives. BMC Endocr Disord. (2013) 13:25. doi: 10.1186/1472-6823-13-25 23885644 PMC3733731

[12] Soufi A Mok E Henderson M Dasgupta K Rahme E Nakhla M . Association of stigma, diabetes distress and self-efficacy with quality of life in adolescents with type 1 diabetes preparing to transition to adult care. Diabetes Med. (2024) 41:e15159. doi: 10.1111/dme.15159 37269172

[13] Howard V Maguire R De Bruin E Deane-King J Duda N Corrigan S . Around-the-clock: Caregiving at night for juveniles living with type 1 diabetes - a systematic review. Psychol Health Med. (2025) 30:1701–22. doi: 10.1080/13548506.2025.2468529 40009726

[14] Cigdem Z Guler S Celik MY . Examining the caregiver burden of parents whose children have type 1 diabetes. J Public Health (Berl). (2023) 31:1523–31. doi: 10.1007/s10389-022-01698-z 30311153

[15] Pomerantz R Williams DD Yeh H-W Clements MA Patton SR . 1453-p: Remedy to diabetes distress (r2d2)—identify the relationship between HbA1c, socioeconomic status (SES), and diabetes distress (DD). Diabetes. (2023) 72:1453–P. doi: 10.2337/db23-1453-P

[16] Rumburg TM Lord JH Savin KL Jaser SS . Maternal diabetes distress is linked to maternal depressive symptoms and adolescents' glycemic control. Pediatr Diabetes. (2017) 18:67–70. doi: 10.1111/pedi.12350 26712240 PMC4927411

[17] Wentzell K Volkening L Laffel L . Concordance and discordance between youth and their parents' reports of diabetes distress: The importance of youth perceptions. J Diabetes Complic. (2022) 36:108207. doi: 10.1016/j.jdiacomp.2022.108207 35610126 PMC10697308

[18] Wyld K Hendrieckx C Griffin A Barrett H D'Silva N . Agenda-setting by young adults with type 1 diabetes and associations with emotional well-being/social support: Results from an observational study. Intern Med J. (2023) 53:1347–55. doi: 10.1111/imj.15919 36008367

[19] Tang TS Seddigh S Halbe E Vesco AT . Testing 3 digital health platforms to improve mental health outcomes in adults with type 1 diabetes: A pilot trial. Can J Diabetes. (2024) 48:18–25:e12. doi: 10.1016/j.jcjd.2023.08.006 37625504

[20] Lohan A Morawska A Mitchell A . A systematic review of parenting interventions for parents of children with type 1 diabetes. Child Care Health Dev. (2015) 41:803–17. doi: 10.1111/cch.12278 26268836

[21] Patton SR Monzon AD Marker AM Clements MA . A nonrandomized pilot of a group, video-based telehealth intervention to reduce diabetes distress in parents of youth with type 1 diabetes mellitus. Can J Diabetes. (2022) 46:262–8. doi: 10.1016/j.jcjd.2021.10.007 35568427 PMC9107594

[22] Garner K Boggiss A Jefferies C Serlachius A . Digital health interventions for improving mental health outcomes and wellbeing for youth with type 1 diabetes: A systematic review. Pediatr Diabetes. (2022) 23:258–69. doi: 10.1111/pedi.13304 34913548

[23] Hood KK Iturralde E Rausch J Weissberg-Benchell J . Preventing diabetes distress in adolescents with type 1 diabetes: Results 1 year after participation in the steps program. Diabetes Care. (2018) 41:1623–30. doi: 10.2337/dc17-2556 29921624 PMC6054495

[24] Shapiro JB Vesco AT Weil LEG Evans MA Hood KK Weissberg-Benchell J . Psychometric properties of the problem areas in diabetes: Teen and parent of teen versions. J Pediatr Psychol. (2018) 43:561–71. doi: 10.1093/jpepsy/jsx146 29267939 PMC6454555

[25] Thompson Burdine J Thorne S Sandhu G . Interpretive description: A flexible qualitative methodology for medical education research. Med Educ. (2021) 55:336–43. doi: 10.1111/medu.14380 32967042

[26] Abadula F Garretson S Okonkwo N LeStourgeon LM Jaser SS . Detangling associations between maternal depressive symptoms and diabetes relationship distress with adolescents’ HbA1c. J Pediatr Psychol. (2023) 49 (2):89–94. doi: 10.1093/jpepsy/jsad070 37794836 PMC10874213

[27] Luo J Wang H Li X Zhou Z Valimaki M Whittemore R . Factors associated with diabetes distress among adolescents with type 1 diabetes. J Clin Nurs. (2021) 30:1893–903. doi: 10.1111/jocn.15742 33829586

[28] Tang TS Sharif N Ng C McLean L Klein G Amed S . Evaluating a digitally delivered, multi-modal intervention for parents of children with type 1 diabetes: A proof-of-concept study. Children (Basel Switzerland). (2024) 11:1114. doi: 10.3390/children11091114 39334646 PMC11430652

[29] Joensen L Fisher L Skinner T Doherty Y Willaing I . Integrating psychosocial support into routine diabetes care: Perspectives from participants at the self-management alliance meeting 2016. Diabetes Med. (2019) 36:847–53. doi: 10.1111/dme.13836 30315608

[30] Perowne R Rowe S Lajevardi A Bingham L Parry E Grey G . Barriers and facilitators to the involvement of under-represented children and young people (aged 8-25) in mental health research - a systematic review. Clin Child Fam Psychol Rev. (2025) 28:858–81. doi: 10.1007/s10567-025-00544-4 40983788 PMC12660437

[31] KL G . Qualitative inquiry with adolescents: Strategies for fostering rich meaning making in group interviews. Am J Qual Res. (2020) 4:52–72. doi: 10.29333/ajqr/8586

[32] Savin-Baden M Major CH . Qualitative research: The essential guide to theory and practice. London: Routledge (2023). p. 608.

