# Supplementary material for: The diabetes distress experience from the perspective of adolescents and their parents
Source: Front Clin Diabetes Healthc. 2026 Jun 11;7:1652578. doi: 10.3389/fcdhc.2026.1652578 (PMC13293899; doi:10.3389/fcdhc.2026.1652578)
Supplement: Supplementary file 1 [file Table1.docx]

Supplementary Material

# Focus group guide: adolescents with T1D

| **Main questions** | **Follow-up points / questions** | **Clarifying questions** |
| --- | --- | --- |
| As a young person living with T1D, what kind of concerns do you experience?  Examples can include anything from interactions with friends and parents, how to manage your diabetes. | - *Social/peer pressure and having T1D (Fitting in and feeling ‘normal’ among peers, not wanting to be treated differently* - *Low motivation and frustration about managing my diabetes 24/7* - *Other medical conditions over and above T1D* - *Worries about my future and future management* - *How to be honest with my parents and health care providers about what I am struggling with* - *How to tell friends, potential love interests, or others that I have T1D* - *How to manage T1D at school or at work* - *Drinking alcohol and using drugs safely with T1D* - *Coping with T1D diagnosis later in life (emotional impact)* - *Feeling like a burden on family and friends* - *Feeling judged if blood sugar is not well managed* - *Diabetes distress and burnout* - *Conflict with parents/care provider about T1D* - *Driving and T1D* | - *Can you tell me a bit more about that?* - *Is there anything else you want to say?* |
| As a young person living with T1D, what kind of emotional or mental health support do you need? Have you needed support in the past? | - *What would that support look like?* - *How would you like to receive support (in person, on the phone, social media, Zoom, other video conference modalities)?* - *To what extent do you prefer individual versus group support?* - *To what extent, if any, have your support needs changed as a result of the COVID crisis* | - *What do you mean about that?* - *Tell me more.* - *Give me an example.* |
| Can you tell me what you think the term diabetes distress means? | - *Have you experienced diabetes distress?* | - Give an example |
| How often, if ever, do you feel overwhelmed or burned out by what you need to do on a daily basis to manage your diabetes? | - *Do you feel this way because you have not been successful with your diabetes management?* - *Do you feel unclear about how to manage your diabetes?* | - What form of help would be useful? |
| How often, if ever, do you feel that those around you act like the diabetes police? | - *How do you feel when others nag you about your diabetes management?* - *Do you feel that your parents think you are incapable of taking care of your diabetes?* | - Is there anything else you want to say? |
| How often, if ever, do you feel that diabetes gets in the way of you having fun and being with your friends? | - *Are there certain activities you feel you are unable to participate in as a result of your diabetes?* - *Do you feel your friends don’t understand how difficult it is to live to diabetes?* | - Do you feel left out? |
| To what extent do you worry about future complications? What about your loved ones (e.g., parents)? | - *Are you constantly concerned about food and eating?* - *Do you worry about getting a low when you are out?* | - What do you use to reassure yourself? |
| If you were to get the support that you needed, what would that look like? |  |  |

# Focus group guide: parents/guardians of adolescents with T1D

| **Main questions** | **Follow-up points / questions** | **Clarifying questions** |
| --- | --- | --- |
| As the parent of an adolescent living with T1D, what kind of worries do you have regarding his/her/their diabetes?  Examples can include anything from interactions with friends, parents of children with T1D, how to manage your child’s diabetes, etc. | - *Social/peer pressure and having T1D (Fitting in and feeling ‘normal’ among peers, not wanting to be treated differently* - *Low motivation and frustration about managing my child’s diabetes 24/7* - *Other medical conditions over and above T1D* - *Worries about my child’s future and future management* - *How to be honest with my and my child’s health care providers about what I am struggling with* - *How to tell friends, potential love interests, or others that my child has T1D* - *How to manage my child’s T1D at school* - *Drinking alcohol and using drugs safely with T1D* - *Coping with T1D diagnosis later in life (emotional impact)* - *Feeling like a burden on family and friends* - *Feeling judged if blood sugar is not well managed* - *Diabetes distress and burnout* - *Conflict with care provider about T1D* - *Driving and T1D* | - *Can you tell me a bit more about that?* - *Is there anything else you want to say?* |
| As the parent of an adolescent with T1D, what kind of emotional or mental health support do you currently need? What do you think your adolescent needs? | - *What would that support look like?* - *How would you like to receive support (in person, on the phone, social media, Zoom, other video conference modalities)?* - *To what extent do you prefer individual versus group support?* - *To what extent, if any, have your support needs changed as a result of the COVID crisis* | - *What do you mean about that?* - *Tell me more.* - *Give me an example.* |
| Can you tell me what you think the term “diabetes distress” means? | - *Have you experienced diabetes distress as the parent?* - *Do you think that your adolescent is experiencing diabetes distress?* | - Give an example |
| How often, if ever, do you feel overwhelmed or burned out by your adolescent’s diabetes regimen? | - *Do you feel this way because your child has not been successful with their diabetes management?* - *Do you feel unclear about how to manage your child’s diabetes?* | - What form of help would be useful? |
| How often, if ever, do you feel that you act like the diabetes police? | - *How do you feel when others nag you about your child’s diabetes management?* - *Do you feel that your child is incapable taking care of their diabetes?* | - Is there anything else you want to say? |
| How often, if ever, do you feel that diabetes gets in the way of your adolescent having fun and being with their friends? | - *Are there certain activities you feel your child is unable to participate in as a result of their diabetes?* - *Do you feel your child’s friends don’t understand how difficult it is to live to diabetes?* | - Do you feel as though your child is left out? |
| How often, if ever, do you worry about future complications? | - *Are you constantly concerned about your child’s food and eating?* - *Do you worry about your child getting a low when you are not around?* | - What do you use to reassure yourself? - What does your child do? |
| If you were to get the support that you needed, what would that look like?  What would the support for your adolescent look like? |  |  |
